# Synthesis and Evaluation of Novel 2-Pyrrolidone-Fused (2-Oxoindolin-3-ylidene)methylpyrrole Derivatives as Potential Multi-Target Tyrosine Kinase Receptor Inhibitors

**DOI:** 10.3390/molecules22060913

**Published:** 2017-05-31

**Authors:** Ting-Hsuan Yang, Chun-I Lee, Wen-Hsin Huang, An-Rong Lee

**Affiliations:** 1 Graduate Institute of Medical Sciences, National Defense Medical Center, No. 161, Section 6, Mingchuan East Road, Taipei 11490, Taiwan; taffw@gmail.com; 2 School of Pharmacy, National Defense Medical Center, No. 161, Section 6, Mingchuan East Road, Taipei 11490, Taiwan; acirlee@gmail.com (C.-I.L.); wenhsin@ndmctsgh.edu.tw (W.-H.H.)

**Keywords:** multi-target kinase inhibitor, VEGFR-2 inhibitor, PDGFRβ inhibitor angiogenesis, (2-oxoindolin-3-ylidene)methylpyrrole

## Abstract

Signaling pathways of VEGFs and PDGFs are crucial in tumor angiogenesis, which is essential in solid tumor progression and metastasis. This study reports our strategy for designing and synthesizing a series of novel 2-pyrrolidone-fused (2-oxoindolin-3-ylidene)methylpyrrole derivatives as potential multi-target tyrosine kinase receptor inhibitors. The target compounds were obtained by condensation of 5-substituted oxindoles with *N*-substituted 2-pyrrolidone aldehyde **7** in satisfactory yields. Of these, **11** and **12** had the highest potency and, compared to sunitinib, showed: (1) significant increase in anti-proliferation of various cancer cells with a favorable selective index (SI); (2) higher inhibitory potency against both VEGFR-2 and PDGFRβ. The molecular modeling results showed that, in terms of VEGFR-2 binding, the synthesized products had a similar binding mode to sunitinib but with tighter interaction.

## 1. Introduction

Angiogenesis, a process of new blood vessel formation from pre-existing vessels, is pivotal in both physiological, such as wound healing, reproduction and embryonic development, and pathophysiological, such as rheumatoid arthritis, atherosclerosis and cancer, conditions [1]. Of the six distinguishing characteristics of cancer, tumor angiogenesis is considered among the most important hallmarks of cancer because it exacerbates malignant cancer growth and metastatic dissemination [2]. To excrete tumor metabolic waste and supply oxygen and nutrients for tumor growth, new blood vessels are essentially formed if the tumor size is beyond 2 mm^3^. To avoid apoptosis, necrosis and/or provide paths for metastasis to survive, tumor cells secrete several pro-angiogenesis factors inducing new blood vessel formation near tumor tissues [3]. These factors include vascular endothelial growth factors (VEGF), platelet-derived growth factors (PDGF), and epidermal growth factors (EGF) [3]. Restricting new blood vessel formation around tumor tissues may also reduce tumor size and/or avoid metastasis and thus bring beneficial effects of targeted therapy.

Many factors, including VEGFs, PDGFs and their receptor tyrosine kinases (RTKs), VEGF receptors 1–3 (VEGFR1–3), and PDGF receptor-α and -β (PDGFR-α/β), participate in tumor-induced angiogenesis signaling pathways in the tumor microenviroment [4]. Among them, VEGFR-1 and -2, key receptors in tumor-induced angiogenic signal transduction, promote endothelial cell proliferation, migration, and survival [3,4,5]. Moreover, PDGFs and PDGFRs support tumor-induced angiogenesis by stimulating proliferation of both fibroblasts and vascular smooth muscle cells, both of which stabilize newly formed blood vessels [5]. Thus, targeting both VEGFs and PDGFs signaling is considered a promising approach to developing anti-angiogenesis drugs.

**Figure 1:**
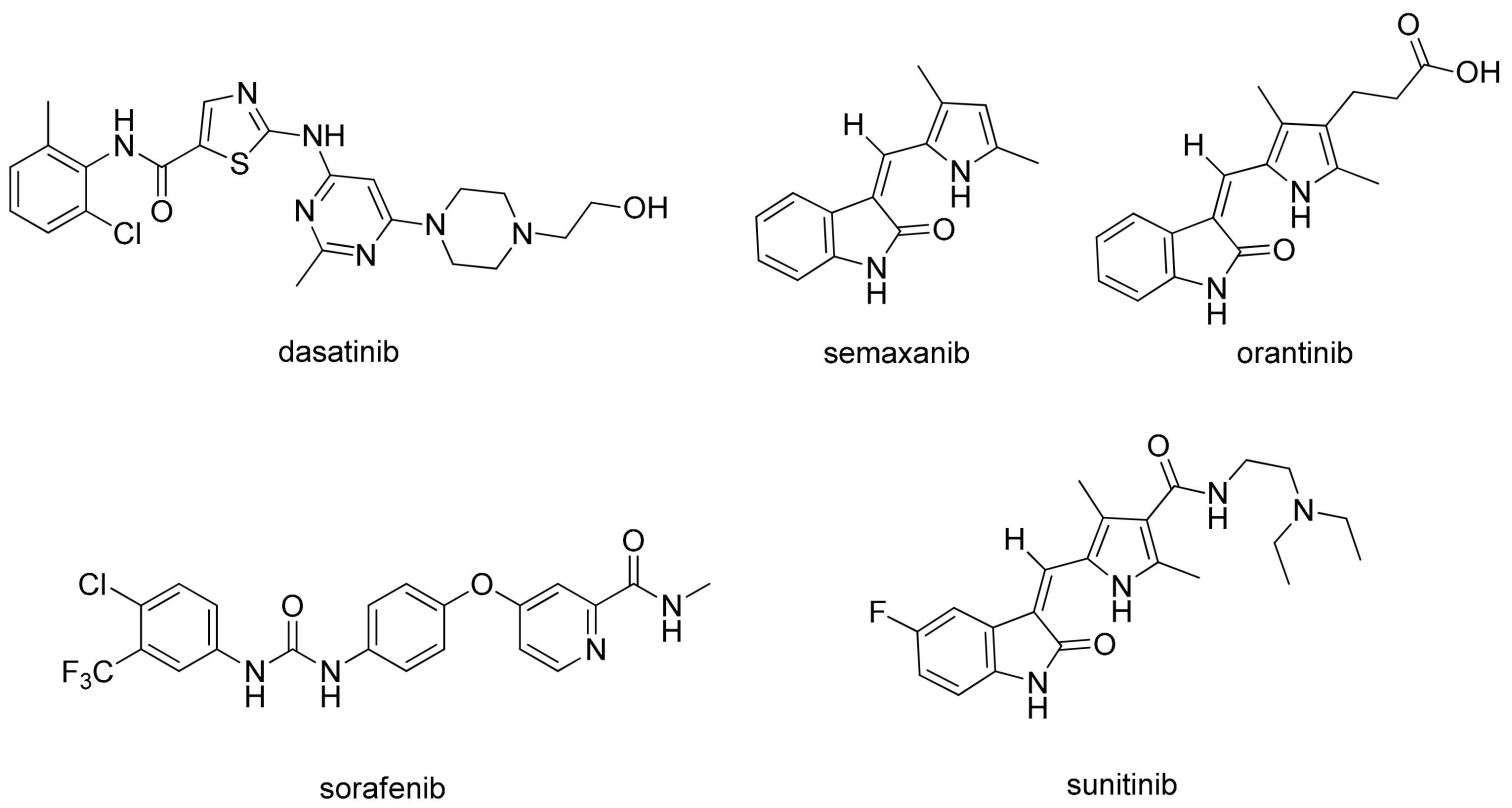
The structures of some multi-targeted kinase inhibitors. Some inhibitors, such as semaxanib, orantinib, and sunitinib, share a common structure of (2-oxindolin-3-ylidene)methylpyrrole nucleus.

In 1971, Folkman proposed that anti-angiogenesis might be an effective strategy for cancer treatment [3]. Sice then, various potent small-molecule ATP-competitive VEGFR inhibitors, such as dasatinib, sorafenib, semaxanib (SU5416), orantinib (SU6668), and sunitinib (Figure 1), have been developed and some of them have been approved [6,7,8,9]. Semaxanib was the first of these to be clinically tested as a receptor tyrosine kinase (RTK) inhibitor for VEGFRs [9]. The lipophilic (2-oxoindolin-3-ylidene)methylpyrrole moiety in semaxanib was then identified as a prerequisite for the inhibitory activity against ATP-competitive RTKs. Further structural modification of semaxanib successfully introduced a substituted pyrrole indolinone derivative sunitinib as a very potent and orally effective anti-angiogenesis agent inhibiting cellular signaling by targeting multiple RTKs, including VEGFRs, PDGFRs, aurora kinases (A, B and C), RET, Flt-3, CSF-1R, and c-Kit [10,11,12,13]. The USFDA approved the use of sunitinib for treating advanced renal cell carcinoma (RCC), gastrointestinal stromal tumors (GISTs), and imatinib-intolerant and -refractory GISTs. Sunitinib was also the first cancer drug simultaneously approved for different indications because of its inhibiting effects in signal pathways that have important roles in both tumor angiogenesis and tumor cell proliferation [14]. Afterwards, multitudinous (2-oxoindolin-3-ylidene)methylpyrrole derivatives have been designed and synthesized as potent small-molecule RTK inhibitors [15,16,17,18,19].

**Figure 2:**
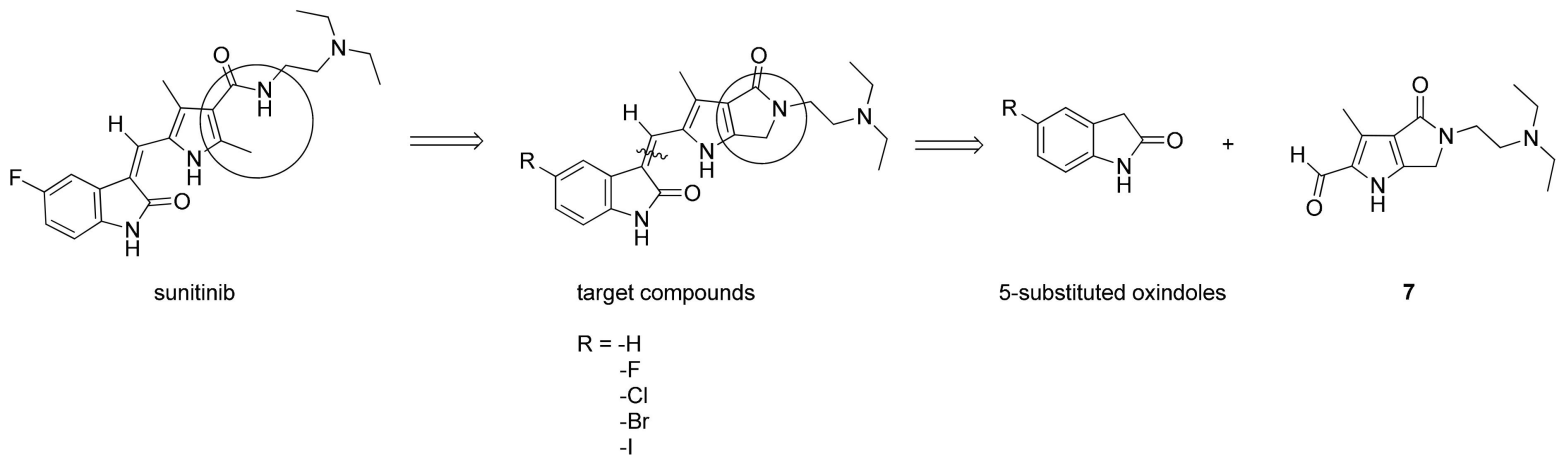
Drug design, and retrosynthetic approach of the target compounds.

Molecular modeling has indicated that the oxindole moiety of sunitinib, crucial for binding to the ATP binding site of RTKs, occupies the adenine pocket and supplies two hydrogen bonds for binding VEGFR2 (Glu917 and Cys919) [20,21]. Additionally, both the pyrrole functionality and the C(5)-F element of (2-oxoindolin-3-ylidene)methylpyrrole in sunitinib structure enhance affinity by inducing further hydrophobic interactions [20]. In contrast, the water-soluble diethylaminoethyl tail of sunitinib lies on the protein surface [19,22,23]. The RTK inhibitory activity and selectivity of (2-oxoindolin-3-ylidene)methylpyrrole derivatives revealed a strong relationships with structural modifications. Jun changed the methylpyrrole part of sunitinib into a bicyclic *N*-substituted pyrrolo-fused six-, seven-, and eight-membered heterocycles to obtain a series of more rigid analogs. They discovered certain products as potent inhibitors of VEGFR-2, PDGFRβ and c-kit both enzymatically and cellularly [19]. This study reports the design, synthesis, and structure-activity relationships (SARs) of a series of novel (2-oxoindolin-3-ylidene)methylpyrrole derivatives, bearing at C(5) of oxindole various halogens, fused with an *N*-substituted 2-pyrrolidone which might result in a smaller, flatter, and more rigid conformation (Figure 2), and thus restrict the ability of the hydrophilic diethylaminoethyl tail to rotate in the hope of facilitating the binding affinity. The target molecules were prepared by condensation of 5-substituted oxindoles with *N*-substituted 2-pyrrolidone aldehyde **7**. Several of the resulting compounds proved to be potent small-molecule kinase inhibitors. Among them, **11** and **12** consistently displayed the most potent activities and favorable selective indexes (SI) and thus were chosen as the candidates for further preclinical development.

## 2. Results and Discussion

### 2.1. Chemistry

**Scheme 1:**
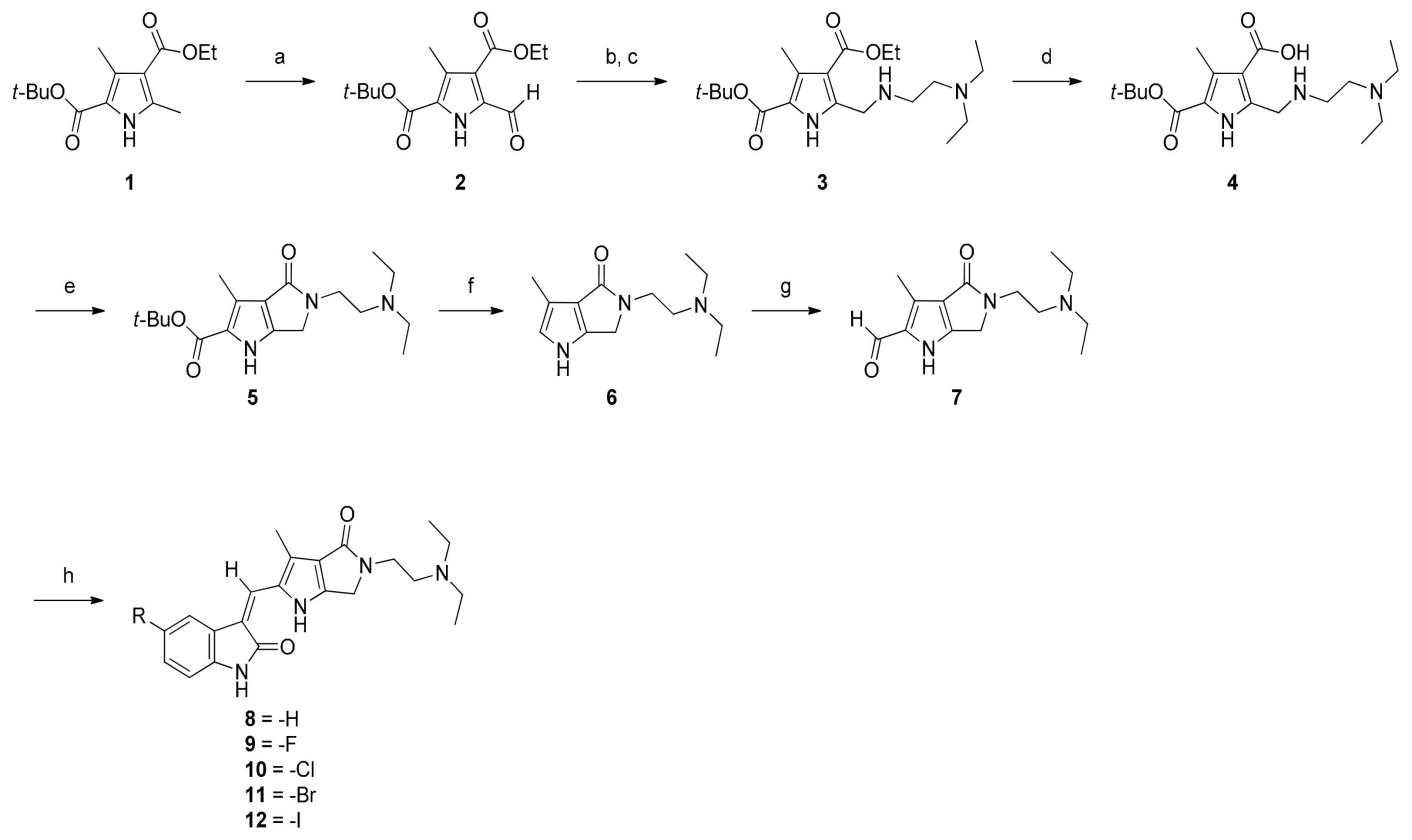
Synthesis of *N*-substituted 2-pyrrolidone fused (2-oxoindolin-3-ylidene)methylpyrrole derivatives. Reagents and conditions: (a) CAN, THF, AcOH, H_2_O, 1 h, 98%; (b) *N*,*N*-diethylethylenediamine, EtOH, r.t., 4 h; (c) NaBH_4_, TsOH H_2_O, 6 h, 90%; (d) 1N NaOH_(aq)_, MeOH, 65 °C, 6 h, 96%; (e) CDI, dry THF, 6 h, 56%; (f) H_2_SO_4_, MeOH, reflux, 4.5 h; (g) Vilsmeier reagent, DCM, r.t., 5 h, 91%; (h) piperidine, indoline-2-ones, EtOH, r.t., 5 h, 43–56%.

Scheme 1 outlines the synthetic sequence that our laboratory used to prepare targets **8**–**12**. The starting material 2-*tert*-butyl 4-ethyl 3,5-dimethyl-1*H*-pyrrole-2,4-dicarboxylate (**1**) was synthesized via the Paal-Knorr pyrrole synthesis [24]. Selective oxidation on α-methyl of **1** with ceric ammonium nitrate (CAN) at room temperature for 1 h quantitatively formed **2**, which bore an α-formyl functionality at the pyrrole ring. Compound **2** was then coupled with *N*,*N*-diethylethylenediamine, and then, without isolation, reduced directly with activated sodium borohydride/*p*-toluenesulfonic acid in ethanol to obtain **3** in a satisfactory yield [25]. Ester **3** was hydrolyzed in methanol into carboxylic acid **4**. Compound **4** was transformed into **5** by lactamization promoted by 1,1′-carbonyldiimidazole (CDI). Hydrolytic decarboxylation of **5** with sulfuric acid in methanol could provide **6**. Without purification, **6** was used directly for selective formylation at C(5) to yield **7**, achieved smoothly by treatment with a Vilsmeier rfeagent. Condensation of **7** with 5-substitued oxindoles in the presence of piperidine at room temperature afforded target molecules.

The key intermediate **3** was initially synthesized using methods described in the literature [25]. First, reductive amination was performed to transform the carbonyl group directly to its corresponding amine derivative **3** by treating aldehyde **2** with *N,N*-diethylethylenediamine followed by *p*-toluenesulfonic acid monohydrate-activated sodium borohydride under solvent-free conditions; however, compound **3** was obtained in only 43% yield, along with a string of unidentifiable impurities. In our modified experiments, ethanol was used as the solvent for reductive amination of **2**. The resulting yield substantially increased to 90%. We hypothesized that the use of ethanol in this reaction vastly improved the yield of **3** because the protic polar solvent ethanol might effectively be involved in the formation of an appropriate transition state required for the subsequent reductive amination and thus greatly facilitated the reaction. The unreacted **2** and all the impurities generated were then easily removed by column chromatography.

**Scheme 2:**
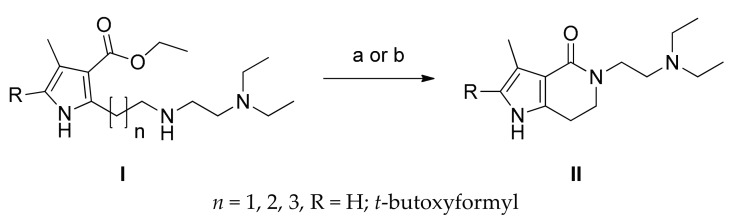
Synthesis of pyrrolo-fused-heterocycles-2-indolinones **II** [19]. Reagents and conditions: (a) LiOH/ethylene glycol; (b) AlMe_3_, toluene, then HCl.

In the synthesis of the first bicyclic compound **5**, Jun revealed that an elegant direct lactamization that formed lactams with medium size (six-, seven-, and eight-membered ring) could be used either by treating **I** with lithium hydroxide or with trimethylaluminium, followed by treating with 2 M hydrochloric acid if decarboxylation products were desired, to give pyrrolo-fused-heterocycles-2-indolinones **II** in the yield of 27–97% (Scheme 2) [19]. Their experimental results showed that lactamization yields correlated with the ring size of the products. The marked success of Jun in synthesizing medium-sized lactams with simultaneous decarboxylation prompted us to prepare the smaller but desired lactam **6**. By applying this seemingly promising approach, our synthesis of **6** would have been achieved in a single step starting from **3**. Unfortunately, all our attempt lactamization of **3**, based on this strategy, to obtain desired **5** under various conditions failed and culminated in producing the hydrolyzed product **4**. Reactions at higher temperatures only gave trace amount of the product. Variation in solvents and use of microwave irradiation usually obtained yields approximating 10%. Even the modified method using DABAL-Me_3_ or AlMe_3_/DABAL-Me_3_ or all other similar approaches under various conditions proved to be fruitless [25,26,27,28,29]. The likely culprit is the significant difference in the ring strain between **6** and **II** (*n* = 1, 2, 3). These experimental results clearly indicate that the Jun’s method of lactamization excludes the preparation of *N*-substituted 2-pyrrolidone derivatives to be used in our synthesis.

Our laboratory finally found a way to circumvent the predicament in a stepwise manner. First, ester **3** was hydrolyzed into carboxylic acid **4** with 1 N sodium hydroxide in 96% yield. Performing CDI-mediated lactamization of the resulting zwitterionic compound **4** in dry tetrahydrofuran under a nitrogen atmosphere proved effective for synthesizing the desired fused pyrrole **5** in 56% yield. Compound **5** was then subjected to hydrolytic decarboxylation with sulfuric acid under a reflux condition to provide **6**. Without isolation and purification, crude **6** was smoothly formylated at C(2) by treatment with a Vilsmeier reagent in dichloromethane to furnish **7** in 91% yield. Condensation of **7** with 5-substitued oxindoles afforded target molecules **8**–**12** in 43–56% yields.

All target compounds were isolated as free bases. In most cases, crystallization or column chromatography was required for purification. The structures of most synthetic intermediates and products were established by spectroscopy and high-resolution mass spectrometry.

### 2.2. Anti-Proliferation Activity and Acute Cytotoxicity

Table 1 shows the in vitro anti-proliferation activity of the synthetic products **8**–**12** against human colon cancer line HCT116, human non-small cell lung cancer cells NCI-H460, and human renal cell carcinoma 786-O. For comparison, sunitinib was employed as a positive control. The preliminary results showed that all synthetized compounds had significant inhibiting effects on HCT-116 cancer cell proliferation, but for **8** which bears C(5)-H. While the IC_50_ values of **9** (C(5)-F) and **10** (C(5)-Cl) were approximately equal to that of sunitinib (IC_50_ 3.42 ± 0.57 μM), the values for **11** (C(5)-Br) (IC_50_ 1.05 ± 0.18 M) and **12** (C(5)-I) (IC_50_ 0.42 ± 0.16 μM) were much better. As to the anti-proliferation effect on the NCI-H460 cells, **8**–**10** showed poor inhibitory activity (IC_50_ > 10 μM); **11** was approximately equal in potency to sunitinib (IC_50_ = 6.57 and 6.23 μM, respectively). Notably, **12** revealed prominent inhibitory activity (IC_50_ = 2.95 μM). As to the anti-proliferation effect on the 786-O cells, both **8** and **9** were once again void of activity (IC_50_ > 10 μM). The potencies of compounds **10**–**12** (IC_50_ approximately 7 μM) were comparable to that of sunitinib.

Potential anticancer drug candidates should have greater selectivity for tumor cells compared with normal cells. Therefore, human normal fibroblast cells Detroit 551 were used for evaluation so as to indicate the selective indexes of **8**–**12** and sunitinib. In Table 1, our synthetic compounds generally showed much less toxicity to Detroit 551 cells than sunitinib. Even potent **12** had high selectivity against HCT116 and NCI-H460 cancer cell lines.

**Figure 3:**
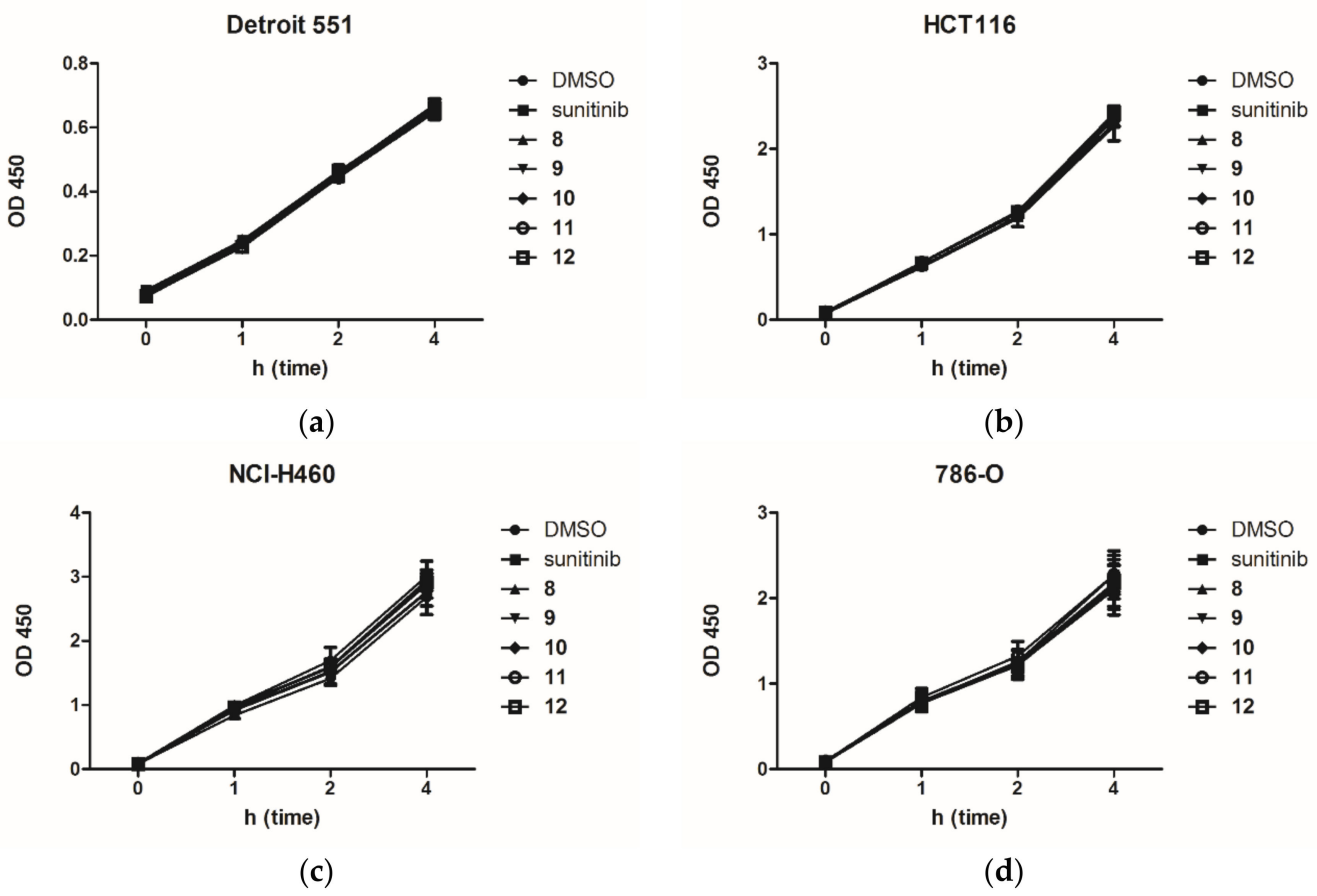
Acute cytotoxicity assay of (**a**) Detroit 551; (**b**) HCT116; (**c**) NCI-H460; (**d**) 786-O cells incubated with DMSO (1%), sunitinib and **8**–**12** (10 μM).

To exclude the possibility that the inhibitory responses of sunitinib and compounds **8**–**12** were resulted from the acute cytotoxicity of indicated compounds, this study performed a WST-8 assay to assess their effects of acute cellular toxicity on Detroit 551, HCT116, NCI-H460 and 786-O cell lines. Figure 3 shows the experimental results. The results clearly revealed that all the synthetic products **8**–**12**, together with sunitinib, were nearly free from acute cellular toxicity on all the tested cell lines.

In the (2-oxoindolin-3-ylidene)methylpyrrole system, the C(5) halogen substituent is known to have critical roles in the cytotoxicity and anti-proliferation of cancer cells [16,18,19,30,31]. Structural modification of sunitinib by replacing the fluorine atom on C(5) with an iodine led to approximately 23-fold weaker potency against HT29 cells [30]. In our 2-pyrrolidone-fused (2-oxoindolin-3-ylidene)methylpyrrole system, compound **9** (C(5)-F) had approximately equal or slightly better potency in the anti-proliferation activity against HCT116 to that of sunitinib (IC_50_ 2.94 μM vs. 3.42 μM). The corresponding **10** (C(5)-Cl), **11**, (C(5)-Br) and **12** (C(5)-I) showed a similar trend. For example, compound **12** was strikingly potent, with approximately 8-fold stronger than sunitinib and 10-fold stronger than **8** (C(5)-H).

Apoptosis of human colon cancer cells, including HCT116 and HT29 cells, can be induced by VEGFR-2 inhibitors, such as regorafenib, crizotinib, sorafenib, and sunitinib, via p53 upregulated modulator of apoptosis (PUMA) [32,33,34,35]. Recently, Zhang demonstrated that PUMA could be transcriptionally activated via the NF-κB pathway after aurora kinase inhibition in HCT116 cells [36]. Sunitinib has well recognized inhibitory activity against aurora kinase, and the six-membered cycloalkanone fused (2-oxoindolin-3-ylidene)methylpyrroles exhibit further improvement in the inhibition of aurora kinase [18]. We hypothesized that our synthetic derivatives might have a profound effect on the binding affinity of aurora kinase. Therefore, an aurora A kinase inhibition percentage at 1.0 μM of sunitinib and the synthetic compounds were thus assessed to validate our hypothesis. Table 1 shows the experimental results, which indicated that all our newly synthetic products showed excellent but without noticeably differentiating inhibitory activity against aurora A (92.1–95.9%), comparing to sunitinib (50.7%). Thus, these results suggested that, in binding to and inhibition of aurora A, compared to the conformation of the bicycle pyrrole scaffold, the C(5) substitution effect might play a less significant role.

**Figure 4:**
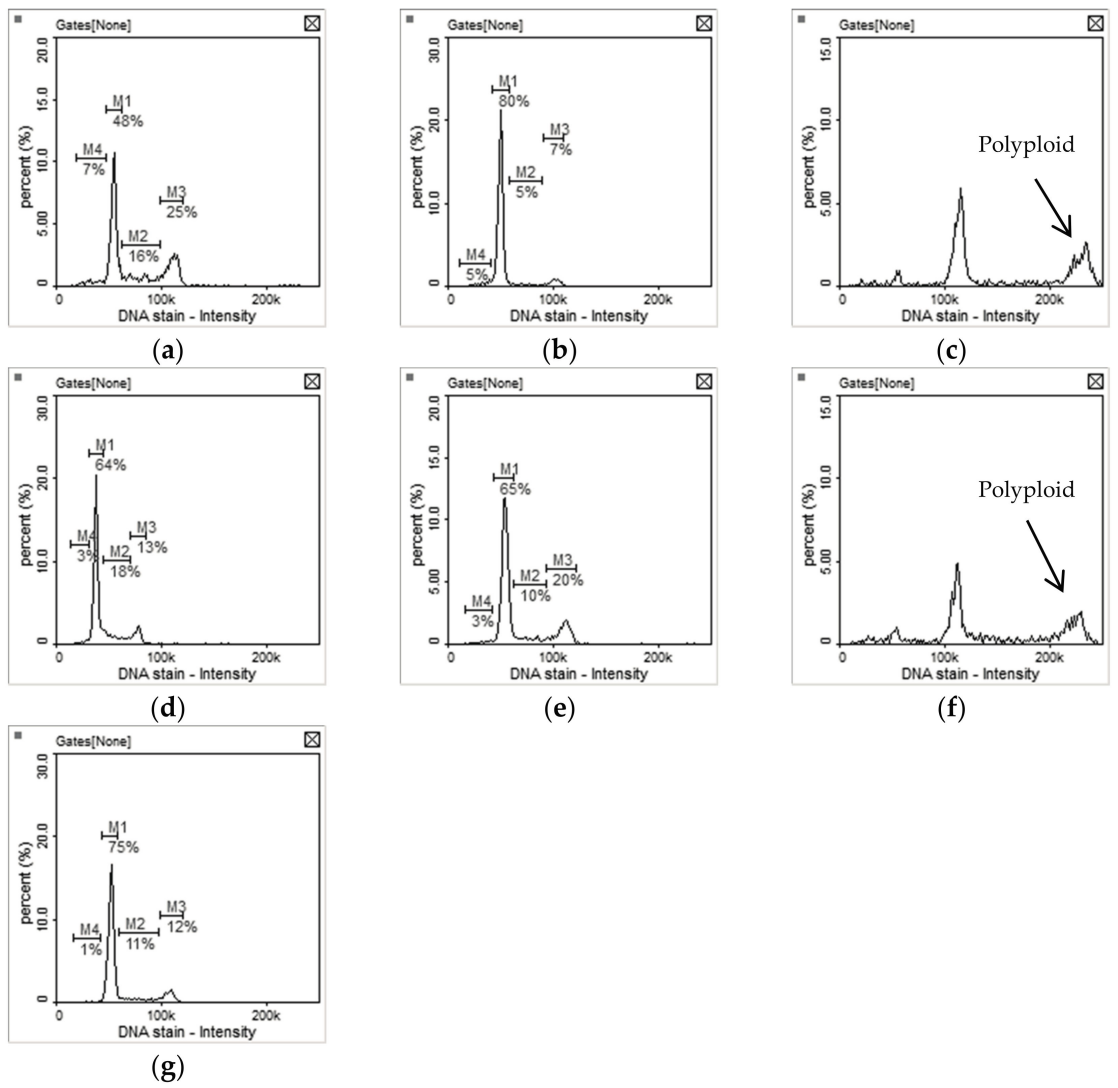
Cell cycle profiles of HCT-116 cells treated with (**a**) 1% DMSO (control); (**b**) 5.0 μM of sunitinib; (**c**) 5.0 μM of **8**; (**d**) 5.0 μM of **9**; (**e**) 5.0 μM of **10**; (**f**) 1.0 μM of **11**; (**g**) 1.0 μM of **12** for 24 h. M1: G0/G1phase, M2: S phase, M3: G2/M phase.

Inhibition of aurora kinase leads to a polyploidy cell cycle profile [18,37]. The cell cycle profile of HCT116 cells incubated with compounds **8** and **11** for 24 h exhibited an increase phase of polyploid (Figure 4c,f). Surprisingly, the cell cycle profile of HCT116 cells incubated with sunitinib, **9**, **10**, and **12** for 24 h caused G0/G1 cell cycle arrest (Figure 4b,d,e,g). The best explanation is that compounds **8** and **11** inhibited HCT116 cells proliferation via significant aurora kinase inhibition while sunitinib, **9**, **11** and **12** might be otherwise involved in different mechanisms.

In summary, the experiments in this study suggest that the structural skeleton constructed by fusion of (2-oxoindolin-3-ylidene)methylpyrrole with *N*-substituted 2-pyrrolidone and introduction of a proper halogen at C(5) of oxindole has a strong influence on both anti-proliferation activity and selectivity. The C(5)-halogen substituents of the title compounds seemed to be advantageous in general and had marked effect: the bulkier halogen the substituent is, the stronger anti-proliferation activity it has.

### 2.3. Kinase Inhibitory Assays

Table 2 shows the results of kinase inhibition assays for compounds **8**-**12**, compared to sunitinib. The IC_50_ values for **8**, **10**, **11**, and, **12** against VEGFR-2 were 4- to 6-fold higher, and their IC_50_ values against PDGFRβ were approximately 2- to 4-fold higher and **11** was the most potent. Comparing with sunitinib, The results once again displayed the similar trend that different C(5) substitutions had significant influence on the biochemical activities against VEGFR-2 and PDGFRβ.

Unexpectedly, comparisons with C(5)-H (**8**) and other C(5)-halogen analogs (**10**, **11**, and **12**) showed that compound **9** (C(5)-F) had much lower inhibitory activity against both VEGFR-2 and PDGFRβ and, comparing to sunitinib, only little increase in inhibitory activity against VEGFR-2 but decrease against PDGFRβ. These results markedly differed from the results reported in experiments using 2-piperidinone fused (2-oxoindolin-3-ylidene)methylpyrrole derivatives [19]. The influence of the difference in ATP binding sites between VEGFR-2 and PDGFRβ is apparent and complex yet nevertheless not very clear. A possible explanation for the increased inhibitory activity of VEGFR-2 is a better accommodation to (2-oxoindolin-3-ylidene)methylpyrroles with C(5)-halides, except for C(5)-F.

### 2.4. In Vitro Tube Formation Assay

**Figure 5:**
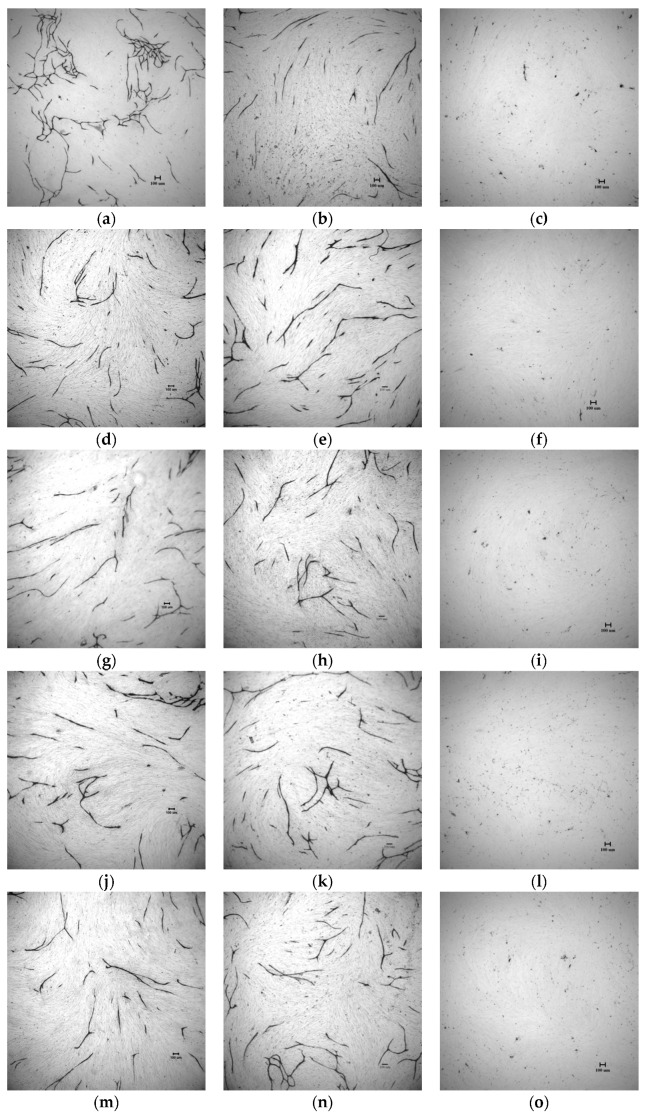

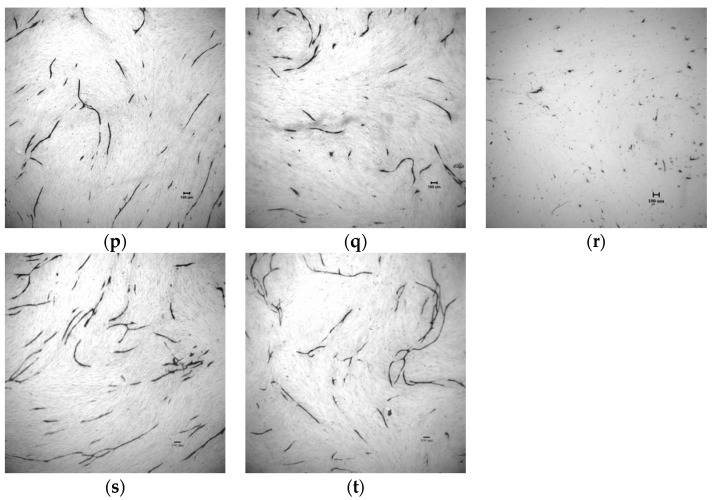
Compounds **8**–**12** inhibited tube formation induced by VEGF. Reprehensive photographs of tube formation (**a**) solvent control; (**b**) VEGFR 10 ng/mL and 70 μM of suramin; (**c**) VEGF 10 ng/mL and 0.50 μM of sunitinib; (**d**) VEGF 10 ng/mL and 0.25 μM of sunitinib; (**e**) VEGF 10 ng/mL and 0.10 μM of sunitinib; (**f**) VEGF 10 ng/mL and 0.50 μM of **8**; (**g**) VEGF 10 ng/mL and 0.25 μM of **8**; (**h**) VEGF 10 ng/mL and 0.10 μM of **8**; (**i**) VEGF 10 ng/mL and 0.50 μM of **9**; (**j**) VEGF 10 ng/mL and 0.25 μM of **9**; (**k**) VEGF 10 ng/mL and 0.10 μM of **9**; (**l**) VEGF 10 ng/mL and 0.50 μM of **10**; (**m**) VEGF 10 ng/mL and 0.25 μM of **10**; (**n**) VEGF 10 ng/mL and 0.10 μM of **10**; (**o**) VEGF 10 ng/mL and 0.50 μM of **11**; (**p**) VEGF 10 ng/mL and 0.25 μM of **11**; (**q**) VEGF 10 ng/mL and 0.10 μM of **11**; (**r**) VEGF 10 ng/mL and 0.50 μM of **12**; (**s**) VEGF 10 ng/mL and 0.25 μM of **12**; (**t**)VEGF 10 ng/mL and 0.10 μM of **12**. Scale bar = 100 μm.

The in vitro inhibitory activity against tube formation of compounds **8**–**12** was tested with an angiogenesis kit (Kurabo). Figure 5 shows that they markedly inhibited the VEGF-induced tube formation at 0.50 μM, 0.25 μM, and 0.10 μM. Enhancement of the inhibitory activity against tube formation with C(5)-halogen substitutions on oxindole followed the order Br > Cl > I > F (Table 3). Compound **9** (C(5)-F) was slightly more potent than sunitinib (*p* < 0.05) and slightly less potent than **8**, C(5) free from halogen substitution; Compound **11** (C(5)-Br) proved to be the most potent with IC_50_ approximately 2-fold stronger than sunitinib (IC_50_ 179 ± 29 nM vs., 387 ± 16 nM). The results were consistent with those for VEGFR-2 kinase inhibitory assays, which suggest that our synthetic compounds inhibit tube formation via inhibiting VEGFR-2 pathway. Therefore, we conclude that compound **11** is a good example of how sunitinib was significantly improved both in inhibiting biochemical activity (VEGFR-2 and PDGFRβ) and in in vitro tube formation via our drug design.

### 2.5. Molecular Modeling

**Figure 6:**
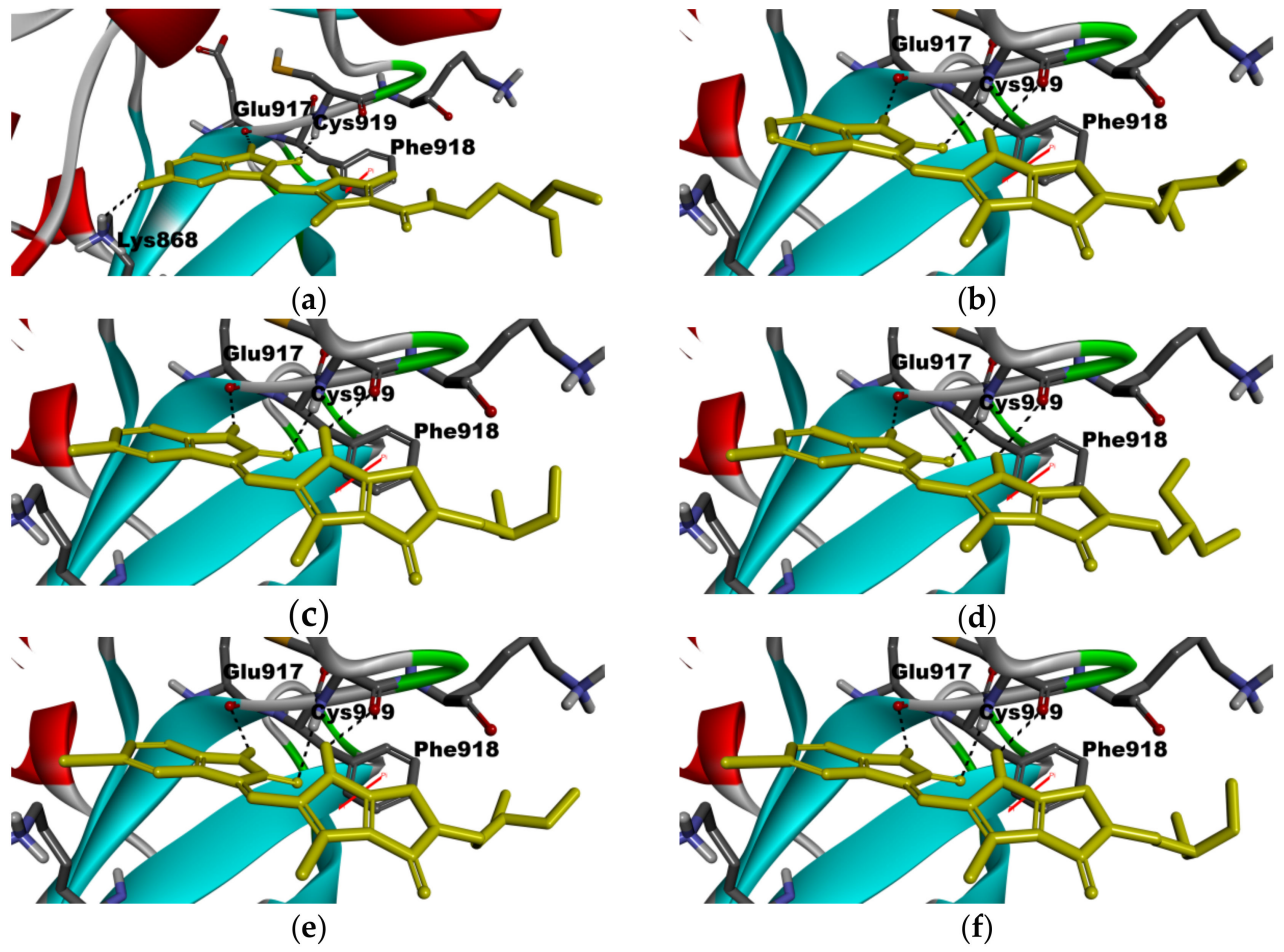
Docked binding modes of **8**–**12** in ATP binding site of VEGFR-2 (PDB ID: 4AGD) and the crystal structure of VEGFR-2 in complex with sunitinib. (**a**) sunitinib (from PDB ID: 4AGD) in VEGFR-2; (**b**) **8** in VEGFR-2; (**c**) **9** in VEGFR-2; (**d**) **10** in VEGFR-2; (**e**) **11** in VEGFR-2; (**f**) **12** in VEGFR-2. Ligands are shown in yellow sticks; hydrogen bonds are shown as dashed black lines; pi-pi interaction is shown in red line.

For a clear understanding of the interaction between the title compounds **8**–**12** together with sunitinib and the ATP-binding site of VEGFR-2 at the molecular level, a molecular modeling was constructed. Figure 6 shows how the test ligands were docked into the ATP-binding site of VEGFR-2 (PDB ID: 4AGD) using Discovery studio LibDock [38]. LibDock is a method that places the generated ligand conformations into the protein active site based on polar and apolar interaction sites (hotspot), and the LibDock score was scored by default settings including Ligscore, piecewise linear potential (PLP), Jain, potential of mean force (PMF) and PMF04. The results showed the predicted binding mode of all our synthetic compounds as well as sunitinib. All our synthetic products and sunitinib formed two hydrogen bonds (important for the binding of (2-oxoindolin-3-ylidene)methylpyrrole derivatives and ATP-binding site) to VEGFR-2, with Glu917 and Cys919, respectively (Figure 6a–f). The pyrrole NH of **8**–**12** formed a hydrogen bond with C=O of Cys919 in VEGFR-2. A hydrophobic interaction also occurred between the pyrrole moieties of **8**–**12** and the phenyl group of Phe918 in VEGFR-2 (Figure 6b–f). Furthermore, the diethylamine tails in **8**–**12** and sunitinib were exposed to the solvent without significant interaction with VEGFR-2. The interaction with VEGFR-2, compounds **8**–**12**, as a whole, showed a similar binding mode to sunitinib but with higher LibDock score (Table 2). Although the LibDock score of **8**–**12** did not show consistent order with the results of **8**–**12** against VEGFR-2, the LibDock score of **8**–**12** were much higher than that of sunitinib (Table 2). However, the interaction was tighter, which might explain why most of our synthetic products exhibited potent activities in the biochemical assay.

## 3. Materials and Methods

### 3.1. Chemistry

All the chemicals were purchased from Aldrich-Sigma Chemical Company (St. Louis, MO, USA), and Alfa-Aesar Chemical Company (Lancashire, Heysham, England) and used without further purification. All reactions were routinely monitored by TLC on Merck F254 silica gel plates. Silica gel (70–230 mesh, Silicacycle) was used for chromatography. The ^1^H- and ^13^C-NMR spectra were determined on an Agilent Varian-400 NMR (Agilent Technologies, Santa Clara, CA, USA) instrument in CDCl_3_, methanol-*d*_4_ or acetic acid-*d*_6_ unless otherwise noted. Chemical shifts (δ) were expressed as parts per million (ppm) downfield from tetramethylsilane (TMS) as the internal standard (σ 0.00), and coupling constants (*J*) were given in hertz (Hz). High-resolution mass spectra (HRMS) using a Bruker Impact HD (ESI) were performed in the Instrument Center of the Ministry of Science and Technology at the National Chiao-Tung University, Taiwan. The reagents 5-chlorooxindole, 5-bromooxindole, and 5-iodiooxindole, used for the synthesis of the target products **10**, **11**, and **12**, respectively, were prepared by the modified methods [39]. Dry tetrahydrofuran was freshly distilled from lithium aluminum hydride (LAH) before use. All the other solvents were obtained from commercial sources and purified before use if necessary. Images were acquired with a Leica DM1000 microscope (Leica Microsystems, Wetzlar, Hessen, Germany). UV-VIS spectra were recorded on a Thermo Multiskan Go Microplate spectrophotometer (Thermo Fisher Scientific, Waltham, MA, USA). IR spectra were registered on a Thermo Nicolet iS5 FT-IR spectrometer (Thermo Fisher Scientific, Waltham, MA, USA) with attenuated total reflection (ATR) method. The purities of the final compounds were greater than 95% as determined by analytical reverse-phase HPLC.

#### 3.1.1. Synthesis of **2**–**7**

##### *5-Formyl-3-methyl-1H-pyrrole-2,4-dicarboxylic acid 2-tert-butyl ester 4-ethyl ester* (**2**)

3,5-Dimethyl-1*H*-pyrrole-2,4-dicarboxylic acid 2-*tert*-butyl ester 4-ethyl ester (**1**) (30.0 g, 0.113 mol) was dissolved in THF (300 mL). To this stirred mixture was added AcOH (360 mL) and water (300 mL) sequentially, then ceric ammonium nitrate (CAN) (246 g, 0.45 mol) was added to the mixture in one portion. After stirring for additional 1 h at room temperature, the reaction mixture was poured into ice-water (1000 mL), and stirred for further 30 min. The precipitate was then filtered and dried in vacuo to yield, after crystallization from EtOAc, 31.1 g of analytically pure **2** (98%) as pale yellow crystals. Mp: 127–128 °C, lit. 120–121 °C [40]. UV λ_max_ (MeOH), nm (logɛ): 238 (3.97). IR (ATR), cm^−1^: 3281, 2994, 1720, 1697, 1660. ^1^H-NMR (400 MHz, CDCl_3_) δ, ppm: 10.25 (s, 1H, C**H**O), 9.79 (s, 1H, N**H**), 4.38 (q, 2H, *J* = 7.2 Hz, -COOC**H_2_**CH_3_), 2.57 (s, 3H, Ar-C**H_3_**), 1.59 (s, 9H, -COOC(C**H_3_**)_3_), 1.26 (t, 3H, *J* = 7.2 Hz, -COOCH_2_C**H_3_**). ^13^C-NMR (100 MHz, CDCl_3_) δ: 182.6, 163.7, 159.8, 132.9, 130.4, 125.1, 120.8, 82.9, 60.8, 28.3, 14.3, 11.3.

##### *2-tert-Butyl-4-ethyl 5-(((2-(diethylamino)ethyl)amino)methyl)-3-methyl-1H-pyrrole-2,4-dicarboxylate* (**3**)

To a stirred solution of 5-formyl-3-methyl-1*H*-pyrrole-2,4-dicarboxylic acid 2-*tert*-butyl ester 4-ethyl ester (**2**) (2.81 g, 10.0 mmol) in EtOH (20 mL) was added dropwise *N*,*N*-diethylethylenediamine (1.5 mL). The resulting mixture was stirred for 4 h at room temperature. Sodium borohydride (0.38 g 10.0 mmol) and toluenesulfonic acid monohydrate (18.0 g, 10.0 mmol) were then added to the mixture, stirred for a further 6 h, quenched with water (150 mL), and extracted with ethyl acetate (30 mL × 3). The combined organic layer was dried over anhydrous Na_2_SO_4_ and filtered. The filtrate was evaporated in vacuo to yield, after column chromatography (silica gel, 90:10:1 EtOAc-MeOH-TEA), 3.4 g (90%) of **3** as pale yellow liquid. UV λ_max_ (MeOH), nm (logɛ): 228 (3.35). IR (ATR) cm^−1^: 3293, 3216, 1685, 1683. ^1^H-NMR (400 MHz, methanol-*d*_4_) δ, ppm: 4.27 (q, 2H, *J* = 7.2 Hz, -COOC**H_2_**CH_3_), 4.05 (s, 2H, Ar-C**H_2_**N-), 2.68 (t, 2H, *J* = 6.0 Hz, -CH_2_NC**H_2_**CH_2_-), 2.55-2.49 (m, 9H, Ar-C**H_3_**, -NC**H_2_**CH_2_N(C**H_2_**CH_3_)_2_), 1.55 (s, 9H, -COOC(C**H_3_**)_3_), 1.34 (t, 3H, *J* = 7.2 Hz, -COOCH_2_C**H_3_**), 1.00 (t, 6H, *J* = 7.2 Hz, -N(CH_2_C**H_3_**)_2_). ^13^C-NMR (100 MHz, CDCl_3_) δ, ppm: 165.5, 161.0, 141.3, 130.2, 119.4, 112.0, 80.9, 59.5, 52.5, 47.4, 47.0, 46.2, 28.4, 14.4, 11.8, 11.5.

##### *2-tert-Butyl 5-(((2-(diethylamino)ethyl)amino)methyl)-3-methyl-1H-pyrrole-2,4-dicarboxylic acid* (**4**)

To a solution of **3** (2.43 g, 6.40 mmol) in MeOH (20 mL) was added dropwise 1N NaOH (20 mL) and the resulting mixture was then heated to reflux for 4 h. After cooling, the mixture was neutralized with 1 N HCl and the solvent was then evaporated under reduced pressure. MeOH (20 mL) was added to the residue and then filtered. The filtrate was evaporated in vacuo to get, after recrystallization from hot MeOH, 2.16 g of analytically pure **4** (96%) as pale yellow crystals. Mp: 119-120 °C. IR (ATR), cm^−1^: 3208, 1568, 1475, 1471, 1137, UV λ_max_ (MeOH), nm (logɛ): 222 (2.98). ^1^H-NMR (400 MHz, methanol-*d*_4_) δ, ppm: 4.36 (s, 2H, Ar-C**H_2_**N-), 3.58 (t, 2H, *J* = 7.0 Hz, -CH_2_NC**H_2_**CH_2_-), 2.69 (t, 2H, *J* = 7.0 Hz, -CH_2_NCH_2_C**H_2_**-), 2.61 (q, 4H, *J* = 7.2 Hz, -N(C**H_2_**CH_3_)_2_), 2.41 (s, 3H, Ar-C**H_3_**), 1.57 (s, 9H, -COOC(C**H_3_**)_3_), 1.04 (t, 6H, *J* = 7.2 Hz, -N(CH_2_C**H_3_**)_2_). ^13^C-NMR (100 MHz, methanol-*d*_4_) δ, ppm: 169.2, 162.5, 146.0, 126.5, 123.5, 121.1, 82.1, 52.1, 48.1, 47.2, 41.1, 28.7, 11.6, 10.9.

##### *5-(2-(Diethylamino)ethyl)-3-methyl-4-oxo-1,4,5,6-tetrahydropyrrolo[3,4-b]pyrrole-2-carboxylic acid tert-butyl ester* (**5**)

Compound **4** (5.44 g, 16.0 mmol) and CDI (5.19 g, 32.0 mmol) were suspended in dry THF (300 mL) under a nitrogen atmosphere, and then heated to 65 °C for 6 h. The reaction mixture was then filtered. The filtrate was evaporated under reduced pressure and the residue was purified to give, after column chromatography (silica gel, 90:10:1 EtOAc-MeOH-TEA), 3.0 g of the bicyclic compound **5** (56%) as pale yellow solids. Mp: 176 °C. UV λ_max_ (MeOH), nm (logɛ): 270 (3.18), IR (ATR), cm^−1^: 3202, 1652, 1682. ^1^H-NMR (400 MHz, methanol-*d*_4_) δ, ppm: 4.38 (s, 2H, ArC**H_2_**N-), 3.61 (t, 2H, *J* = 6.9 Hz, -NC**H_2_**CH_2_-), 2.72 (t, 2H, *J* = 6.9 Hz, -NCH_2_C**H_2_**-), 2.67 (q, 4H, *J* = 7.2 Hz, -N(C**H_2_**CH_3_)_2_), 1.07 (t, 6H, *J* = 7.2 Hz, -N(CH_2_C**H_3_**)_2_). ^13^C-NMR (100 MHz, CDCl_3_) δ, ppm: 166.5, 161.7, 143.2, 125.3, 122.9, 121.7, 81.4, 52.0, 47.1, 46.4, 40.6, 28.4, 11.8, 10.6.

##### *5-(2-(Diethylamino)ethyl)-3-methyl-4-oxo-1,4,5,6-tetrahydropyrrolo[3,4-b]pyrrole-2-carbaldehyde* (**7**)

To a solution of **5** (1.34 g, 4.00 mmol) in MeOH (100 mL) was added dropwise 15% H_2_SO_4_ (10 mL). The resulting mixture was then heated to reflux for 4.5 h. After the reaction mixture was cooled, the solution was adjusted to pH 14 with 6 N NaOH and extracted with ethyl acetate (30 mL × 3). The organic layers were combined and then dried over anhydrous Na_2_SO_4_ to obtain crude compound **6** which was used for the next step without further purification. To a solution of crude **6** (1.21 g, 5.10 mmol) in dichloromethane (15 mL) was added Vilsmeier reagent (0.82 g, 6.1 mmol). The reaction mixture was allowed to stir at room temperature for 5 h and then was adjusted to pH 14 by 1 N NaOH and extracted with ethyl acetate (10 mL × 3). The organic layers were combined and dried over anhydrous Na_2_SO_4_ and evaporated to give, after column chromatography (silica gel, 90:10:1 EtOAc-MeOH-TEA), 1.1g of **7** (91%) as pale yellow solids. Mp: 170–171 °C. UV λ_max_ (MeOH), nm (logɛ): 221 (2.92). IR (ATR), cm^−1^: 3093, 2702, 1639, 1562. ^1^H-NMR (400 MHz, CDCl_3_) δ, ppm: 9.20 (s, 1H, C**H**O), 4.36 (s, 2H, Ar-C**H_2_**-), 3.56 (t, 2H, *J* = 6.4 Hz, -CH_2_NC**H_2_**CH_2_-), 2.64 (t, 2H, *J* = 6.4 Hz, -CH_2_NCH_2_C**H_2_**-), 2.55 (q, 4H, *J* = 7.2 Hz, -N(C**H_2_**CH_3_)_2_), 2.47 (s, 3H, Ar-C**H_3_**), 1.00 (t, 6H, *J* = 7.2 Hz, -N(CH_2_C**H_3_**)_2_). ^13^C-NMR (100 MHz, CDCl_3_) δ, ppm: 178.4, 165.8, 148.1, 134.1, 128.5, 121.9, 51.6, 46.6, 46.3, 40.4, 11.3, 8.9.

#### 3.1.2. Synthesis of **8**–**12**

General procedure for the preparation of compounds **8**–**12**.

To each stirred solution containing compound **7** (263 mg, 1.0 mmol) in EtOH (15 mL) was added dropwise a solution of 5-substituted oxindole (1.0 mmol) in EtOH (2 mL) and then piperidine (0.1 mL). After stirring at room temperature for 6 h, the precipitate formed was filtrated, washed with EtOH, and purified by column chromatography (silica gel, 90:10:1 EtOAc-MeOH-TEA).

*(Z)-3-((5-(2-(Diethylamino)ethyl)-3-methyl-4-oxo-1,4,5,6-tetrahydropyrrolo[3,4-b]pyrrol-2-yl)methylene)indolin-2-one* (**8**). Yield: 50%, pale orange solids. Mp: 167–168 °C. UV λ_max_ (MeOH), nm (logɛ): 390 (4.12), IR (ATR), cm^−1^: 3172, 1651, 1568. ^1^H-NMR (400 MHz, acetic acid-*d*_4_) δ, ppm: 7.75 (s, 1H, -C=C**H**-), 7.73 (d, 1H, *J* = 7.6 Hz, Ar**H**), 7.18 (dd, 1H, *J* = 7.6, 7.6 Hz, Ar**H**), 7.03 (dd, 1H, *J* = 7.6, 7.6 Hz, Ar**H**), 6.98 (d, 1H, *J* = 7.6 Hz, Ar**H**), 4.59 (s, 2H, Ar-C**H_2_**-), 3.55 (t, 2H, *J* = 6.4 Hz, -NC**H_2_**CH_2_-), 2.65 (t, 2H, *J* = 6.4 Hz, -NCH_2_C**H_2_**-), 2.55 (q, 4H, *J* = 7.2 Hz, -N(C**H_2_**CH_3_)_2_), 2.47 (s, 3H, Ar-C**H_3_**), 0.99 (t, 6H, *J* = 7.2 Hz, -N(CH_2_C**H_3_**)_2_), ^13^C-NMR (100 MHz, acetic acid-*d*_4_) δ, ppm: 171.5, 170.0, 149.6, 139.3, 133.6, 126.3, 126.0, 125.2, 123.4, 120.9, 119.6, 118.6, 111.4, 51.9, 48.6, 48.2, 39.6, 9.9, 8.8. HRMS *m/z* (ESI): calcd., 379.2129 [M + H]^+^_,_ found, 379.2133 [M + H]^+^.

*(Z)-3-((5-(2-(Diethylamino)ethyl)-3-methyl-4-oxo-1,4,5,6-tetrahydropyrrolo[3,4-b]pyrrol-2-yl)methylene)-5-fluoroindolin-2-one* (**9**). Yield: 56%, orange solids. Mp: 245–246 °C, UV λ_max_ (MeOH), nm (logɛ): 291 (4.33). IR (ATR), cm^−1^: 3534, 3400, 1668, 1572 cm^−1^. ^1^H-NMR (400 MHz, acetic acid-*d*_4_) δ, ppm: 7.62 (s, 1H, -C=C**H**-), 7.38 (dd, 1H, *J* = 9.0, 2.0 Hz, Ar**H**), 6.96–6.90 (m, 2H, Ar**H** × 2), 4.59 (s, 2H, Ar-C**H_2_**-), 3.97 (t, 2H, *J* = 6.0 Hz, -NC**H_2_**CH_2_-), 3.49 (t, 2H, *J* = 6.0 Hz, -NCH_2_C**H_2_**-), 3.37 (q, 4H, *J* = 7.2 Hz, -N(C**H_2_**CH_3_)_2_), 2.47 (s, 3H, Ar-C**H_3_**), 1.31 (t, 6H, *J* = 7.2 Hz, -N(CH_2_C**H_3_**)_2_). ^13^C-NMR (100 MHz, acetic acid-*d*_4_) δ, ppm: 169.8, 166.1, 158.6, 148.3, 135.5, 132.4, 127.1, 125.9, 125.0, 121.0, 117.1, 113.7, 110.7, 107.0, 49.0, 46.7, 46.6, 37.3, 10.0, 8.7. HRMS *m/z* (ESI): calcd., 397.2034 [M + H]^+^; found, 397.2038 [M + H]^+^.

*(Z)-5-Chloro-3-((5-(2-(diethylamino)ethyl)-3-methyl-4-oxo-1,4,5,6-tetrahydropyrrolo[3,4-b]pyrrol-2-yl)methylene)indolin-2-one* (**10**). Yield: 54%, orange solids. Mp: 252–253 °C, UV λ_max_ (MeOH), nm (logɛ): 393 (4.37). IR (ATR), cm^−1^: 3162, 1634, 1582 cm^−1^. ^1^H-NMR (400 MHz, acetic acid -*d*_4_) δ, ppm: 7.64 (s, 1H, -C=C**H**-), 7.62 (d, 1H, *J* = 2.0 Hz, Ar**H**), 7.16 (dd, 1H, *J* = 8.2, 2.0 Hz, Ar**H**), 6.96 (d, 1H, *J* = 8.2 Hz, Ar**H**), 4.59 (s, 2H, Ar-CH_2_), 3.97 (t, 2H, *J* = 6.0 Hz, NC**H_2_**CH_2_-), 3.49 (t, 2H, *J* = 6.0 Hz, -NCH_2_C**H_2_**-), 3.37 (q, 4H, *J* = 7.2 Hz, -N(C**H_2_**CH_3_)_2_), 2.47 (s, 3H, Ar-C**H_3_**), 1.31 (t, 6H, *J* = 7.2 Hz, -N(CH_2_C**H_3_**)_2_). ^13^C-NMR (100 MHz, acetic acid-*d*_4_) δ, ppm: 171.2, 169.7, 149.9, 137.7, 133.6, 128.6, 128.5, 127.9, 127.4, 126.6, 121.1, 119.6, 117.2, 112.5, 51.8, 48.6, 48.1, 39.5, 9.9, 8.7. HRMS *m/z* (ESI): calcd., 413.1744 [M + H]^+^; found, 413.1743 [M + H]^+^.

*(Z)-5-Bromo-3-((5-(2-(diethylamino)ethyl)-3-methyl-4-oxo-1,4,5,6-tetrahydropyrrolo[3,4-b]pyrrol-2-yl)methylene)indolin-2-one* (**11**). Yield: 56%, orange solids. Mp: 266–267 °C. UV λ_max_ (MeOH), nm (logɛ): 394 (4.56), IR (ATR), cm^−1^: 3161, 1634, 1581 cm^−1^. ^1^H-NMR (400 MHz, acetic acid -*d*_4_) δ, ppm: 7.74 (d, 1H, *J* = 1.6 Hz, Ar**H**), 7.59 (s, 1H, -C=C**H**-), 7.30 (dd, 1H, *J* = 8.4, 1.6 Hz, Ar**H**), 6.90 (d, 1H, *J* = 8.4 Hz, Ar**H**), 4.55 (s, 2H, Ar-C**H_2_**-), 3.96 (t, 2H, *J* = 6.0 Hz, -NC**H_2_**CH_2_-), 3.50 (t, 2H, *J* = 6.0 Hz, -NCH_2_C**H_2_**-), 3.38 (q, 4H, *J* = 7.2 Hz, -N(C**H_2_**CH_3_)_2_), 2.46 (s, 3H, Ar-C**H_3_**), 1.33 (t, 6H, *J* = 7.2 Hz, -N(CH_2_C**H_3_**)_2_). ^13^C-NMR (100 MHz, acetic acid-*d*_4_) δ, ppm: 171.0, 169.6, 149.8, 138.0, 133.6, 130.7, 128.4, 127.3, 126.4, 122.4, 121.1, 117.0, 115.8, 112.9, 51.8, 48.5, 48.1, 39.5, 9.9, 8.7. HRMS *m/z* (ESI): calcd., 457.1239 [M + H]^+^; found, 457.1236 [M + H]^+^.

*(Z)-3-((5-(2-(Diethylamino)ethyl)-3-methyl-4-oxo-1,4,5,6-tetrahydropyrrolo[3,4-b]pyrrol-2-yl)methylene)-5-iodoindolin-2-one* (**12**). Yield: 43%, brown solids, Mp: 267–268 °C. UV λ_max_ (MeOH), nm (logɛ): 394 (4.36), IR (ATR), cm^−1^: 3174, 1689, 1584 cm^−1^. ^1^H-NMR (400 MHz, acetic acid -*d*_4_) δ, ppm: 7.94 (s, 1H, -C=C**H**), 7.64 (s, 1H, Ar**H**), 7.50 (d, 1H, *J* = 8.0 Hz, Ar**H**), 6.81 (d, 1H, *J* = 8.0 Hz, Ar**H**), 4.59 (s, 2H, Ar-C**H_2_**-), 3.97 (t, 2H, *J* = 6.0 Hz, -NC**H_2_**CH_2_-), 3.49 (t, 2H, *J* = 6.0 Hz, -NCH_2_C**H_2_**-), 3.37 (q, 4H, *J* = 7.2 Hz, -N(C**H_2_**CH_3_)_2_), 2.47 (s, 3H, Ar-C**H_3_**), 1.31 (t, 6H, *J* = 7.2 Hz, -N(CH_2_C**H_3_**)_2_). ^13^C-NMR (100 MHz, acetic acid-*d*_4_) δ, ppm: 171.6, 169.8, 149.9, 149.9, 149.8, 133.5, 127.5, 126.5, 121.1, 116.1, 114.6, 112.2, 112.1, 106.7, 51.6, 49.4, 48.2, 39.5, 9.9, 8.8. HRMS *m/z* (ESI): 505.1100 [M + H]^+^; found, 505.1094 [M + H]^+^.

### 3.2. Biology

#### 3.2.1. Cell Culture

The HCT116 (human colon cancer cells, BCRC 60349) and Detroit 551 (human normal fibroblast cells, BCRC 60118) were maintained in DMEM (Gibco, Grand Island, NY, USA) containing 10% FBS (HyClone, Logan, UT, USA). NCI-H460 (BCRC 60373) and 786-O (BCRC 60243) were maintained in RPMI 1640 (Gibco, Grand Island, NY, USA) containing 10% FBS (HyClone, Logan, UT, USA). They were incubated at 37 °C in a 5% CO_2_ atmosphere.

#### 3.2.2. Cell Proliferation Assay

The cells incubated as above were plated at a density of 2000 cells/well (cancer cells) [18,41,42] or 10,000 cells/well (Detroit 551) on a 96-well plate for 24 h; serial dilutions of indicated compounds were added and incubated for additional 72 h. At the end of the incubation, cell viability was determined by 3-(4,5-dimethylthiazol-2-yl)-2,5-diphenyl tetrazolium bromide (MTT). The MTT formazan crystals were dissolved by DMSO, and the absorbance at 570 nm using a microplate spectrophotometer (Thermo Fisher Scientific, Waltham, MA, USA) [43].

#### 3.2.3. Acute Cytotoxicity

The acute cytotoxicity effect of sunitinib and compounds **8**–**12** was determined by Cell-Counting-Kit-8 (Dojindo, Rockville, MD, USA) assay on HCT116, NCI-460, 786-O and Detroit 551 with the manufacturer’s protocol. Cells were seeded at 5000 cells/well on a 96-well plate for 24 h. The indicated compounds in different concentration (100 μL) were added to cells. Post 6 h, old medium was aspirated, and cells were washed three times with PBS. WST-8 (Dojindo, Rockville, MD, USA) (10 µL) was added to each well, and the absorbance of the plate was recorded at 450 nm on a microplate spectrophotometer (Thermo Fisher Scientific, Waltham, MA, USA).

#### 3.2.4. Image Cytometry

Cell cycle profiles of HCT116 cells were determined with an NC-3000 image cytometer (ChemoMetec, Allerod, Denmark) in accordance with manufacturer’s protocol. Briefly, cells were seeded at 200,000 cells/well on a 6-well plate for 24 h. 2 mL of indicated compounds (5.0 μM for sunitinib and **8**–**10**, 1.0 μM for **11** and **12**) were added to cells. After incubation for 24 h, 100,000 cells were harvested and centrifuged for 5 min at 400 g at room temperature, washed once with PBS (50 μM) and resuspended with 50 μL of lysis buffer (ChemoMetec, Allerod, Denmark) containing 10 μg/mL of 2-(4-amidinophenyl)-1*H*-indole-6-carboxamidine (DAPI, ChemoMetec, Allerod, Denmark). The cells were incubated at 37 °C for 5 min, 50 μL of stabilization buffer (ChemoMetec, Allerod, Denmark) was added to the solution, and the cellular fluorescence was measured with an NC-3000 image cytometer using NC-SlideA8 (ChemoMetec, Allerod, Denmark). The NC-3000 software (ChemoMetec, Allerod, Denmark) was used for image acquisition, image analysis and quantification and data visualization.

#### 3.2.5. In Vitro Tube Formation Assay

Angiogenesis Kit (Kurabo, Neyagawa, Osaka, Japan) was used in accordance with the manufacturer’s protocols. Briefly, HUVECs and fibroblasts were co-cultured in 24-well plates with basal medium or solvent control medium (basal medium containing 1.0% DMSO) or solvent control medium containing **9**, **10**, **11**, and **12** (0.50 μM, 0.25 μM and 0.10 μM). The medium renewals with or without agent in cultures were conducted every 3 days. After 11 days HUVECs were fixed and stained with anti-CD31 antibody in line with the manufacturer’s protocols. The tube formation area were measured using Angiogenesis Image Analyzer (Kurabo, Neyagawa, Osaka, Japan) with suaramin (70 μM) as positive control.

#### 3.2.6. In Vitro Kinase Assay

The Reaction Biology Corporation (http://www.reactionbiology.com) HotSpot assay platform was used to determine the inhibitory activity of compounds **8**–**12** and sunitinib against VEGFR-2, PDGFR and aurora A, which was measured by quantifying the amount of 33P incorporated into the substrate in the presence of the test compound [44]. Briefly, specific kinase and substrate and required cofactors were prepared in reaction buffer. Test compounds were added into the reaction, after 20 min, a mixture of ATP (Sigma, St. Louis, MO, USA) and ^33^P ATP (Perkin Elmer, Waltham, MA, USA) was added to make a final concentration of 10.0 μM. Reactions were stood at room temperature for 120 min, and spot the reactions onto P81 ion exchange filter paper (Whatman Inc., Piscataway, NJ, USA). Unbound phosphate was removed by extensive washing of filters in 0.1% phosphoric acid. Kinase activity data was reported as the percent remaining kinase activity in test compounds compared to solvent control (DMSO).

### 3.3. Molecular Modeling

Ligands-receptor docking calculation was carried out using the LibDock protocol. Briefly, receptor active site and ligands were characterized into polar and apolar hotspots. The ligand poses were placed into the receptor site by hotspots accordance. In this study CHARMm force field was used for energy minimization of the ligand molecules and ligand-receptor binding. The binding sphere was defined based on the PDB definition. Conformations of ligands were generated by the BEST method. The LibDock score was scored by default settings including Ligscore, PLP, Jain, PMF and PMF04.

### 3.4. Statistical Analysis

Statistical calculations were carried out with GraphPad Prism version 5. Results were reported as the mean ± SD. Statistical significance was determined by the unpaired student’s *t* test.

## 4. Conclusions

A series of novel *N*-substituted 2-pyrrolidone fused (2-oxoindolin-3-ylidene)methylpyrrole derivatives **8**–**12** were successfully prepared and examined for their anti-tumor activities. Several of them significantly improved inhibitory activity against VEGFR-2 and PDGFRβ over sunitinib. Compared to sunitinib, all our synthetic compounds showed a similar but tighter binding mode with VEGFR-2. The nature of C(5) halogens and the rigid conformation of the scaffold obviously were important determinants of both enzymatic and cellular anti-tumor activities. Among the products obtained, **11** (C(5)-Br) and **12** (C(5)-I) consistently displayed the most potent and favorable selective indexes and might be promising to be used as novel anti-angiogenic agents in the targeted therapy of cancer. The results gleaned from this investigation have important potential applications in the development of new anticancer drugs.

## Figures and Tables

**Table 1:**
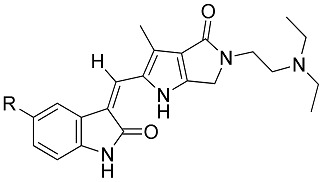
Cellular activities and selective indexes of **8**–**12** and sunitinib.

Compd.	R	IC_50_ (μM)/Selective Index (SI)			
		HCT116	NCI-H460	786-O	Detroit 551
**Sunitinib**	-	3.42 ± 0.57	6.23 ± 0.57	6.27 ± 0.67	9.48 ± 0.18
		2.77	1.52	1.51	
**8**	-H	4.25 ± 1.88	>10	>10	>10
		>2.35	nd	nd	
**9**	-F	2.94 ± 0.66	>10	>10	>10
		>3.4	nd	nd	
**10**	-Cl	3.09 ± 0.70	>10	7.30 ± 1.20	>10
		>3.23	nd	>1.37	
**11**	-Br	1.05 ± 0.18	6.57 ± 1.64	7.06 ± 0.30	>10
		>9.52	>1.52	>1.42	
**12**	-I	0.42 ± 0.16	2.95 ± 0.83	7.76 ± 0.29	9.21 ± 1.61
		21.93	3.12	1.19	

nd: not detected.

**Table 2:**
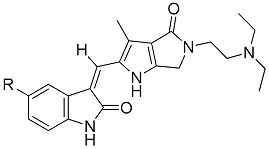
Enzymatic activities and LibDock score of **8**–**12** and sunitinib.

Compd.	R	% Inhibition at 50 nM		IC_50_ (nM)		% Inhibition of Aurora A at 1.0 μM	LibDock Score of VEGFR-2 Binding
		VEGFR-2	PDGFRβ	VEGFR-2	PDGFRβ		
**Sunitinib**	-	29	44	140.0	61.4	50.7	110.303
**8**	-H	56	64	30.2	22.4	95.9	134.754
**9**	-F	35	36	110.7	97.5	93.1	137.901
**10**	-Cl	58	64	35.0	24.9	93.7	130.921
**11**	-Br	64	68	24.7	16.1	93.1	129.433
**12**	-I	58	62	35.1	29.3	92.9	125.031

**Table 3:**
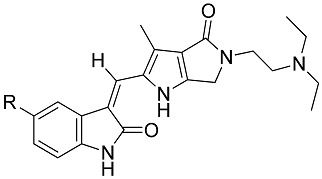
IC_50_ of in vitro VEGF-induced tube formation by **8**–**12** and sunitinib.

Compd.	R	IC_50_ of Tube Area (nM)
**Sunitinib**	-	387 ± 16
**8**	-H	310 ± 15
**9**	-F	367 ± 16
**10**	-Cl	299 ± 70
**11**	-Br	179 ± 29
**12**	-I	313 ± 37
